# Size Scaling in Western North Atlantic Loggerhead Turtles Permits Extrapolation between Regions, but Not Life Stages

**DOI:** 10.1371/journal.pone.0143747

**Published:** 2015-12-02

**Authors:** Nina Marn, Tin Klanjscek, Lesley Stokes, Marko Jusup

**Affiliations:** 1 Division for Marine and Environmental Research, Rudjer Boskovic Institute, Zagreb, Croatia; 2 Southeast Fisheries Science Center, National Marine Fisheries Service, Miami, Florida, United States of America; 3 Faculty of Sciences, Kyushu University, Fukuoka, Japan; CNRS, FRANCE

## Abstract

**Introduction:**

Sea turtles face threats globally and are protected by national and international laws. Allometry and scaling models greatly aid sea turtle conservation and research, and help to better understand the biology of sea turtles. Scaling, however, may differ between regions and/or life stages. We analyze differences between (i) two different regional subsets and (ii) three different life stage subsets of the western North Atlantic loggerhead turtles by comparing the relative growth of body width and depth in relation to body length, and discuss the implications.

**Results and Discussion:**

Results suggest that the differences between scaling relationships of different regional subsets are negligible, and models fitted on data from one region of the western North Atlantic can safely be used on data for the same life stage from another North Atlantic region. On the other hand, using models fitted on data for one life stage to describe other life stages is not recommended if accuracy is of paramount importance. In particular, young loggerhead turtles that have not recruited to neritic habitats should be studied and modeled separately whenever practical, while neritic juveniles and adults can be modeled together as one group. Even though morphometric scaling varies among life stages, a common model for all life stages can be used as a general description of scaling, and assuming isometric growth as a simplification is justified. In addition to linear models traditionally used for scaling on log-log axes, we test the performance of a saturating (curvilinear) model. The saturating model is statistically preferred in some cases, but the accuracy gained by the saturating model is marginal.

## Introduction

Sea turtles face threats globally, and most species are listed by the IUCN (International Union for Conservation of Nature) as threatened, endangered, or critically endangered (see [1] for details), and are protected by national and international laws, such as CITES Convention (Appendix I), Endangered Species Act (ESA, PL93-205), and the Bern convention (CETS No. 104).

The conservation and research of sea turtles are greatly aided by allometry: the study of the relationship of body size to shape, anatomy, physiology, and behavior. Applications of allometry include relating metabolic rate, dive depth and duration, or reproductive output either to body mass [2, 3], or to carapace length [4, 5]. Carapace length is the measurement most often used to report growth rate (in *cm*.*yr* ^− 1^), or construct growth models [6, 7], and age-at-length or life history stage duration models for sea turtles [8–11]. When using carapace length as a measurement of body size, isometric growth is indirectly assumed. Isometric growth implies that ratios of length, width, and body depth are preserved, while size changes during ontogeny or evolution [12], i.e., growth appears to be accompanied with no change in shape.

Allometric scaling is also applied in morphometrics, when noting relative growth rates of different components of the organism [13]. For example, scaling equations are used to convert known measures of sea turtle size to those needed for a specific application (curved carapace length to straight carapace length, carapace length to carapace width etc.). Important application of the conversion equations is in the conservation of sea turtles: for example, dimensions of turtle excluder devices (TEDs) depend on projected turtle carapace width and height. TEDs are openings in fishing nets implemented to reduce the by-catch of sea turtles in shrimp trawls operating inshore and offshore in west Atlantic and east Pacific ocean [14–16]. Turtles inhabiting those areas differ in sizes and life stages, and measuring all dimensions of all life stages in all habitats is impractical, if not impossible. Because in most cases only carapace length is reported, it is important to know relationships between the length and other dimensions of the carapace, and whether or not these relationships differ between areas and/or life stages.

Wallace et al. [3] found that scaling of metabolic rates with mass in three species of sea turtles is allometric, and differs depending on the life stage of the individuals. Differences related to size and/or developmental stage may also be possible in morphology: tail elongation in maturing sea turtle males is a well known example of change in morphological scaling used as an indication of maturation. Sea turtles inhabit a wide range of habitats during their life cycle, so changes in morphology could be related not only to the reproductive functionality (function of tail during copulation), but also as a response to morphological functionality: avoiding predators [17, 18], or adapting shape to new hydrodynamic conditions when changing habitats.

Another source of variability in morphometry, and consequently scaling relations, might be the geographical region. For example, loggerhead turtles in the Mediterranean sea are generally smaller than loggerhead turtles of the same life stage in the North Atlantic ocean [19, 20]. Even though there are cases when the turtles from these regions are grouped together for analysis (e.g. [21, 22]), they are usually studied separately. Geographic subsets of these populations (loggerheads caught in the same area), on the other hand, are sometimes considered morphologically similar and analyzed together. Groups are often arbitrarily defined ([23], but see [24] for list of subpopulation designators), extrapolations are made from one geographic subset to the whole population [14], and scaling models are reported for a population rather than a subset of that population [22, 25, 26]. This approach is practical because identifying the exact region of origin can be done only by genetic analysis [27] as individuals from the same subpopulation may be encountered in different geographical areas [23], and individuals from different subpopulations use the same geographical area [24]. However, the reported (inconclusive) regional differences in scaling relationships between two geographic subsets of adults inhabiting North Atlantic [28], if confirmed, might imply that arbitrary grouping and extrapolations between stages and regions might not be appropriate.

If the understanding of morphology is considerably wrong, the scaling models and management decisions based on such models will be wrong. Differences between geographic subsets would require adaptations in the scaling models and decisions dependent upon size and shape (e.g., TED regulations). Differences between life stages would require adaptations in those decisions based on population models indirectly assuming isometric growth and using only one measure of size (i.e., length) as a proxy for growth or the age of sea turtles. In some cases, specific scaling relationships are not available, and general models must be used for legislation, conservation, or research purposes. If there are considerable errors that arise from utilizing a non-specific relationship, the errors have to be identified and taken into account. Caution has been suggested when extrapolating scaling models to sea turtles of different sizes or from different regions [28, 29]. However, we are not aware of a publication that points to all of the implications, specifically reports morphological scaling relationships, and systematically compares them across regions and life stages.

We focused on the western North Atlantic population of loggerhead turtles. We studied morphometric scaling relationships within two geographic subsets and three life stages of this population. Data collection and types of analyses are described in the first and second part of the methods section. Analyses of two geographic subsets of populations and of three life stage subsets are described in the subsections of results. In the analysis of life stage subsets, we additionally tested the performance of a saturating model described in the section Models and statistical analysis. Results suggest that for a single life stage, data from different geographic regions of western North Atlantic can be modeled together. Young loggerhead turtles that have not yet recruited to neritic habitats (posthatchlings and oceanic juveniles) should be modeled separately from neritic juveniles and adults if accuracy is important. Regardless of differences between life stages, one set of scaling models described the whole size span (multiple life stages) satisfactorily, and produced a good fit when a linear model was fitted on log-log axes. Implications of the results are discussed in the last section of the paper.

## Methods

### Data

We surveyed literature reporting morphometric relationships and/or paired measurements of straight carapace length (SCL), straight carapace width (SCW), and body depth (BD) of North Atlantic loggerhead turtles. We chose to work with straight carapace length (SCL) because measurements of SCL exhibit less variability than those of curved carapace length [29, 30]. Using available resources [28, 31–33]), we gathered data for western North Atlantic loggerhead turtles ranging in SCL from 3.4 cm to 109 cm. Based on the size span of loggerhead turtles [7], and the size range of the data, all post-embryonic life stages were represented. A total of *N* = 17731 data points were obtained, but not all data points were used in the analysis (Table 1).

**Table 1:** Data overview. We studied straight carapace length (SCL), straight carapace width (SCW), and body depth (BD). We used (*SCL*,*SCW*), (*SCL*,*BD*), and (*SCW*,*BD*) data pairs for the analysis, meaning that one data triplet yielded 3 data pairs. See text for details. Life stage subsets: ‘I’—posthatchlings and oceanic juveniles, ‘II’—neritic juveniles, and ‘III’—nesting adults. Range of SCL and SCW values is expressed in cm.

*Type of data*		*All data points*	*Analysis: geographic subsets*		*Analysis: life stage subsets*		
			‘north’	‘south’	‘I’	‘II’	‘III’
(1) (*SCL*, *SCW*) pair	*N =*	371* ^(1,2,3)^	112 ^(3)^	105 ^(2)^	48 ^(1)^	71 ^(1)^	252 ^(1,2,3)^
	SCL range	8.1–109	80.7–107.4	81–109	8.1–41.3	42.1–80.6	80.7 -109
(2) (*SCL*, *BD*) pair	*N =*	280* ^(1,3)^	-	-	55 ^(1)^	71 ^(1)^	154 ^(1,2,3)^
	SCL range	8.1–109			8.1–40.9	41.7–80.6	81.4–109
(3) (*SCW*, *BD*)pair	*N =*	253** ^(1,3)^	-	-	47 ^(1)^	59 ^(1)^	147 ^(1,2,3)^
	SCW range	6.8–98.5			6.8–33.1 ^§^	33.8–61 ^§^	61.5–98.5
(4) (*SCL*, *SCW*, *BD*) triplet	*N =*	5609 ^(4)^	1267	1300 ^†^	1065 ^†^	-	-
	SCL range	3.4–10.1	3.4–10	4.1–10	3.4–10		
*Total number*	*of data pairs*	17731	2646	2705	3345	201	553

**Fig 1:**
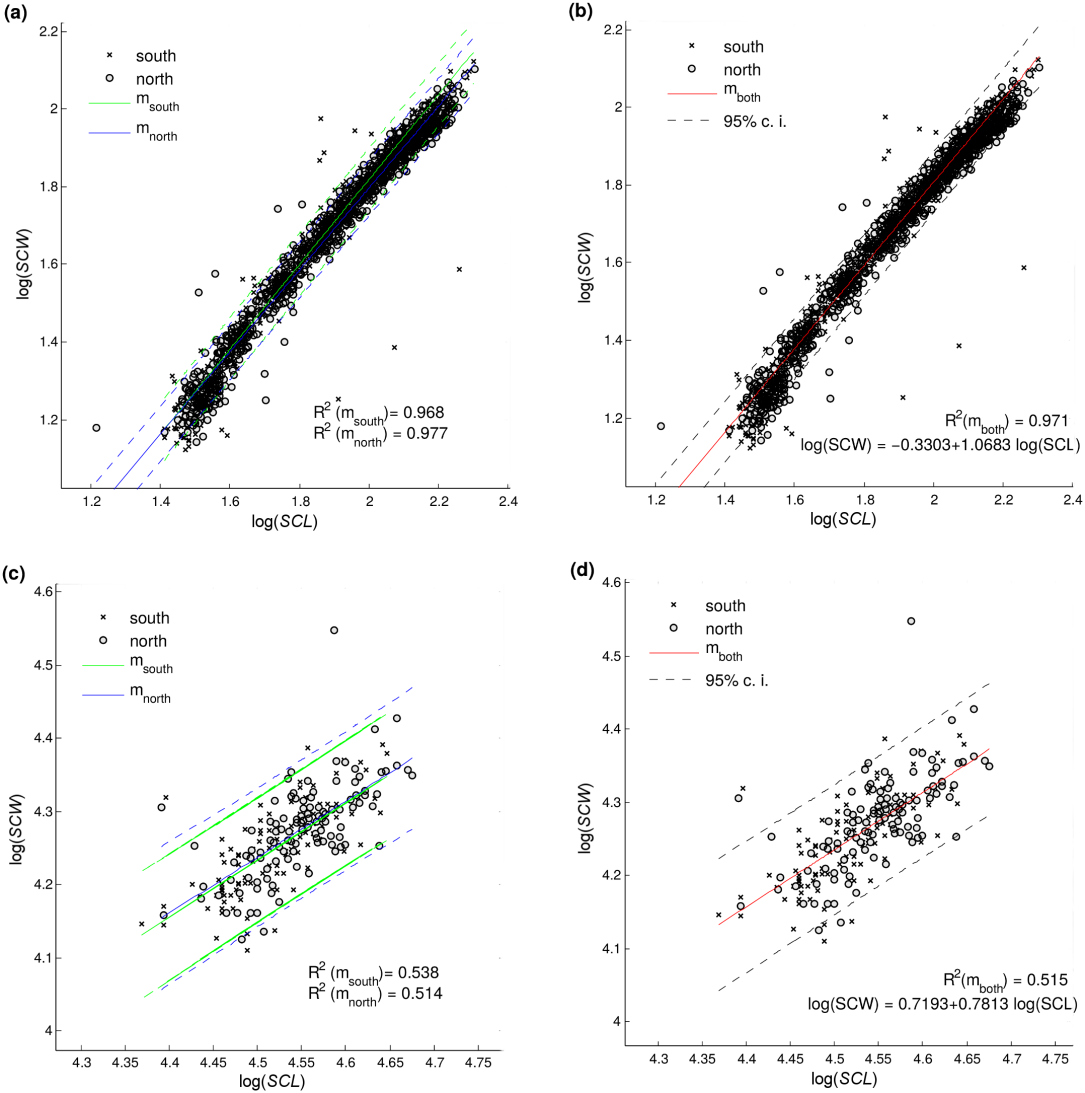
Predictions of log(*SCW*) from log(*SCL*) by regression equations ‘*m*_*north*_’, ‘*m*_*south*_’, and ‘*m*_*both*_’ specific for regional subsets ‘north’, ‘south’, and ‘both’. Panels (a) and (b): data from the posthacthling group. Panels (c) and (d): data for the adult group. The recommended regression equations are displayed in the plot, while the parameters for remaining equations are provided in Table 3. Dashed lines mark the 95% confidence intervals of the predictions.

**Fig 2:**
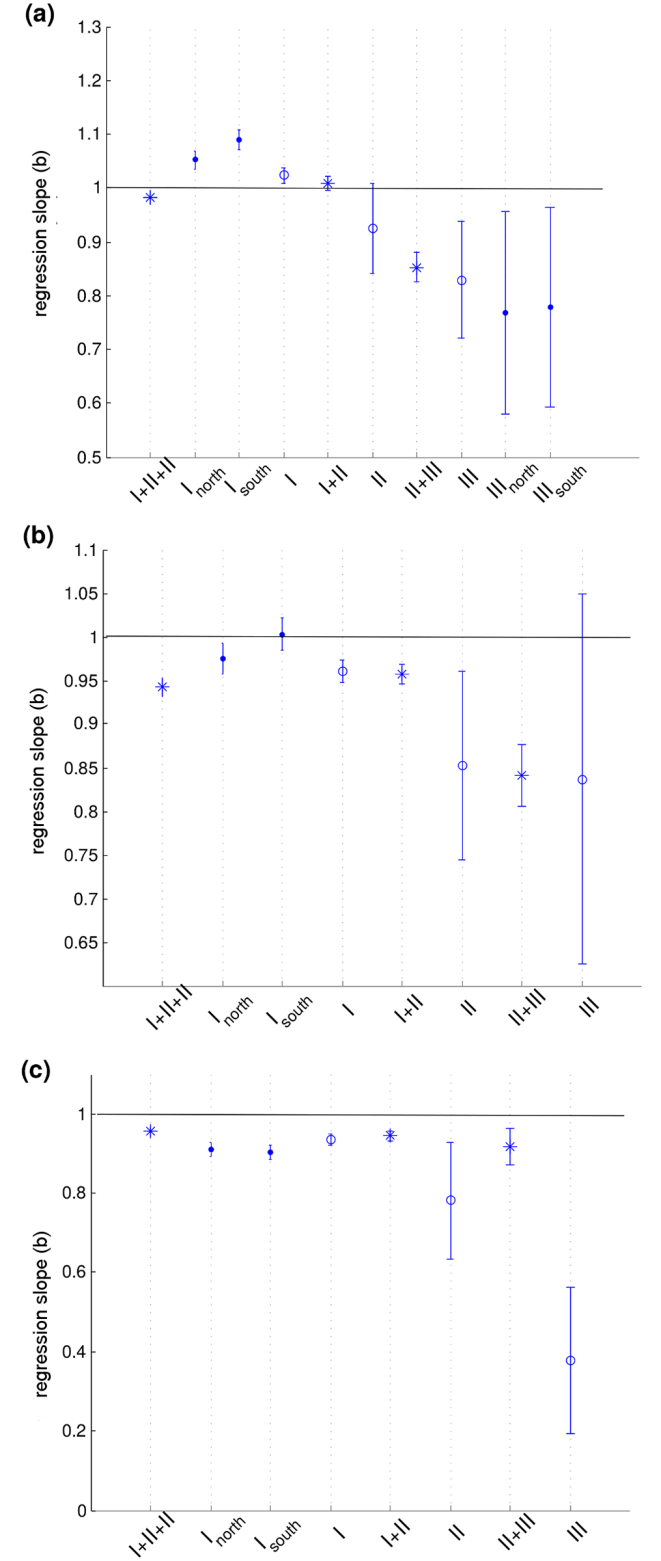
Model slopes with 95% confidence intervals. For scaling relationships of SCW to SCL (panel (a)), BD to SCL (panel (b)), and BD to SCW (panel (c)). In addition to slopes of regression equations specific for life stage subsets (‘I’, ‘II’, and ‘III’, marked with circles), and combined data sets (‘I + II’, ‘II + III’, and ‘I + II + III’, marked with asterisks), we show slopes of regression equations specific for regional subsets, which describe either exclusively posthatchlings (‘*I*_*north*_’, ‘*I*_*south*_’) or nesting adults (‘*III*_*north*_’, ‘*III*_*south*_’), all marked with dots. Analysis of regional subsets is described in section Analysis of regional subsets ‘north’ and ‘south’. Horizontal full line represents the slope of an isometric model (*b* = 1).

Data sources:

^1^[32], Figs 1 and 2 from Appendix 1;

^2^[31], Fig 3;

^3^[28], Fig 2 panels c and d;

^4^ this study

* Digitalization software PlotReader (version 1.55.0.0) was used for data import. Overlaping datapoints could not be differentiated.

** Data pairs reconstructed by relating (log(*SCL*), log(*SCW*)), and (log(*SCW*), log(*BD*)) data pairs, by using common values of log(*SCL*). In cases where there was more than one value from one relationship mapping to the single value of the other (due to overlap of the data points), the average of the values was paired with the common measurement.

^§^ For this relationship, the SCW to SCL relationship was used for dividing data into subsets: smallest SCW from subset ‘II’ was used as SCW at recruitment, and smallest SCW from subset ‘III’ was used as SCW at nesting

^†^ data censored to perform a more balanced analysis: 1300 data triplets were randomly chosen from 4342 data triplets available for that population subset, to match the number of data triplets for the other subset. Later, 1056 data triples were randomly chosen from the 2567 triplets, so that the percentage of (SCL, SCW) data pairs of subset ‘I’ matches the percentage of the total size span occupied by this subset.

For the first analysis, we grouped the data into two subsets, based on the geographic region where turtles were encountered and measured: northern subset (‘north’) consisting of sea turtles that hatched or were found nesting in South Carolina, and southern subset (‘south’) consisting of sea turtles that hatched or were found nesting in the area around Florida peninsula. Data triplets (Table 1) were raw data for captive reared posthatchlings (up to 10 weeks old), and data pairs were data points digitized from graphed logarithmic relationships of SCW to SCL, and BD to SCL for wild nesting adults. For the purposes of this analysis, we assumed that the relationship of carapace length and carapace width is not affected by captive rearing conditions, only the rate at which the turtles reach a certain size. However, considering other factors (life stage, type of data), we decided to analyze posthatchlings and nesting adults separately. In the posthatchling group, there was almost three times as much data for the ‘south’ than for the ‘north’ subset, so data from the ‘south’ were censored (randomly selected 1300 triplets) to match the number of data triplets of the ‘north’ subset. Consequently, we used a total of 2567 data triplets for posthatchlings (analyzed as SCW to SCL, BD to SCL, and BD to SCW data pairs), and 227 SCW to SCL data pairs for adults (Table 1).

For the second analysis, we divided the data into three subsets: ‘I’, ‘II’, and ‘III’, based on the length of individuals. Each of the three subsets represented a different life stage: ‘I’—young loggerhead turtles that have not recruited to neritic habitat (posthatchlings and oceanic juveniles), ‘II’—neritic juveniles, and ‘III’—nesting adults. We used 41.5 cm SCL as a size at recruitment to neritic habitat, and 80.7 cm SCL as a size at onset of nesting (becoming an adult). Carapace length of a sea turtle at the time of recruitment is between 41.5 and 58.2 cm SCL (converted from 46 cm and 64 cm CCL reported in [9]). Although [34] report a narrower range (48.5–51.5cm SCL), we conservatively used the lower end of the wider range reported in [9]. Size at onset of nesting was determined as the minimum reported for nesting females [28, 31]. Using the lower end was a conservative estimate to ensure that sea turtles that have already recruited to neritic habitats, or have started nesting, are not grouped and analyzed with those who have not. Subset ‘I’ initially had 89% of the data points, even though it covers only 38% of the total SCL range in the data. To avoid giving too much weight to the subset, and achieve a more uniform distribution of datapoints across the size span, we sub-sampled the subset ‘I’ in such a way that the relative number of data pairs in subset ‘I’ of the SCW to SCL relationship reflected the 38% calculated for the SCL span: 2567 data triplets used in the previous analysis were additionally censored to 1065, and then used to construct SCW to SCL, BD to SCL, and BD to SCW data pairs. Next, data from other sources were added. Type, number and sources of data points are given in Table 1.

In addition to data sets listed in Table 1, we created a dataset ‘both’ for the analysis of combined geographic subsets, and datasets ‘I + II’, ‘II + III’, and ‘I + II + III’ for the analysis of combined life stage subsets. The additional data sets were merged combinations of the censored subsets.

### Models and statistical analysis

To test specificity of allometric scaling relationships of western North Atlantic loggerhead turtles, we performed two analyses: (i) analysis of geographic subsets (‘north’ and ‘south’), and (ii) analysis of life stage subsets (‘I’, ‘II’, ‘III’). Each analysis consisted of two steps.

In the first step, for each subset we calculated pairwise ratios *r*_1_, *r*_2_, and *r*_3_ between the three variables (straight carapace length—*SCL*, straight carapace width—*SCW*, and body depth -*BD*): *r*_1_ = *SCW*/*SCL*, *r*_2_ = *BD*/*SCL*, *r*_3_ = *BD*/*SCW*. In the analysis of geographic subsets (‘north’ and ‘south’), we could calculate ratios *r*_1_, *r*_2_, and *r*_3_ for posthatchlings, and only ratio *r*_1_ for adults in each subset. In the analysis of life stage subsets (‘I’, ‘II’, ‘III’), we could calculate ratios *r*_1_, *r*_2_, and *r*_3_ for each of the three subsets.

We compared the ratios by plotting their distributions, and calculating standard descriptive statistics (median, interquartile range, min, max). By analyzing the ratios, we obtained a first glance at the differences and/or similarities among the compared groups. For example, the distribution of the ratios between two regions should stay the same if animals from those regions have similar shapes. Additionally, the analysis of the ratio distributions between life stages highlighted the extent to which loggerhead turtles deviate from the assumption of isomorphism.

In the second step, the power law was used to scale *SCW* to *SCL*, *BD* to *SCL*, and *BD* to *SCW*. The power law,

$$y=A\cdot x^{b},$$

where *A* is the conversion factor from one characteristic to another, and *b* defines the nature of the scaling (isometric if *b* = 1, allometric otherwise), was found to describe a multitude of correlations between size and metabolic activity or behavior, and was also applied in morphometrics [12, 13].

We first log_*e*_ transformed the data to reduce the effect of outliers, stabilize variance, and linearize the relationship for least squared-error regression [35]. The log transformation of the power law resulted in three linear models, one for each scaling relationship:

log(SCW)=a+b·log(SCL), (1)

log(BD)=a+b·log(SCL), (2)

log(BD)=a+b·log(SCW), (3)

where *a* is log_*e*_(*A*), the intercept on the y-axis, and b is the slope of the line on the log-log plot. The linear models (Eqs 1–3) were fitted to log_*e*_ transformed data described in section Data using least squared-error linear regression (fit function implemented in MATLAB R2011b). We evaluated the goodness of fit (coefficient of determination *R* ^2^), and used analysis of covariance (ANOCOVA) models and multiple comparison procedures to compare the model slopes with ANCOVA and Tukey-Kramer test (*p* < 0.05, aoctool and multcompare functions implemented in MATLAB R2011b).

In the analysis of regional data sets (‘north’, ‘south’, and ‘both’), we fitted models (1) to (3) to each data set within the posthatchling group, and model (1) to data sets within the adult group. Results are given in the section Analysis of regional subsets ‘north’ and ‘south’.

In the analysis of life stage data sets (‘I’, ‘II’, ‘III’, ‘I + II’, ‘II + III’, and ‘I + II + III’), we fitted models (1) to (3) to each data set. While comparing life stage subsets, we could not test for differences between regional subsets within each life stage. Therefore, data obtained from different geographic regions were pooled for analysis. The pooling is further justified by the observed uniformity of carapace length and width among nesting loggerheads from different western North Atlantic subpopulations ([36], and references within). The uniform distribution of data points (achieved by censoring the subset ‘I’ prior to the analysis) made it possible to estimate model parameters on merged groups, without attributing too much weight to any of the stages. For the models fitted on all available data (the combined dataset ‘I + II + III’), we tested whether the growth of sea turtles is isometric, by testing whether the parameter *b* is significantly different from 1. We calculated what would the covariant variables (log(*SCW*) and log(*BD*)) be for a given value of log(*SCL*) or log(*SCW*) if growth is isometric, using as input the average SCL and SCW values at hatching, recruitment, and nesting. We compared the predictions by the isometric model (*b* = 1) to the predictions by the allometric model (*b* regressed by model fitting), calculated the error, and compared the prediction intervals of the models. Results are given in the section Analysis of life stage subsets ‘I’, ‘II’, and ‘III’.

The preliminary results suggested a non-linear relationship of the data on the log-log axes. Therefore, we also investigated whether a curvilinear model would perform significantly better. We chose a type II functional form of the saturating relationship because it had the same number of parameters as the linear model. Other models, for example those suggested by [37, 38], might have been equally appropriate, but they would either introduce new parameters that do not add to the mechanistic explanation [37], or would require fitting using untransformed data, thereby obstructing direct comparison of parameter values [38]. The type II functional form of the saturating relationship was:

$$y=\frac{A\cdot x}{b+x}.$$

After log transformation, we got:

log(SCW)=a+log(SCL)-log(SCL+b), (4)

log(BD)=a+log(SCL)-log(SCL+b), (5)

log(BD)=a+log(SCW)-log(SCW+b). (6)

We compared the performance of linear models (Eqs 1–3, marked with ‘M1’), and non-linear models (Eqs 4–6, marked with ‘M2’) using goodness of fit statistics (*R* ^2^ and RMSE), and Akaike Weights [39]. The goodness of fit statistics evaluate model performance independently: higher *R* ^2^, and lower RMSE (Root Mean Square Error) indicate better performance. The Akaike weight for a certain model is a probability that the particular model is the best model of those investigated, given the particular data [39]. Akaike weights, therefore, cross-compare the performance of the models by evaluating the probability with which one model should be chosen over the other. Results are given in the section Performance of saturating models ‘M2’. All calculations were done in MATLAB R2011b.

## Results

Coefficients of scaling relationships between length, width, and depth of western North Atlantic loggerhead turtles did not significantly differ between two regional subsets, but were significantly different between life stage subsets. When accuracy is not of paramount importance, the common model for all life stages can be used, and isometric growth can be assumed. The tested non-linear models, although statistically preferred over the linear class of models in some cases, did not yield considerably different results.

### Analysis of regional subsets ‘north’ and ‘south’

Each regional subset (‘north’ and ‘south’) of western North Atlantic loggerheads consisted of a posthatchling and an adult group, which were analyzed separately (see subsection for details). The standard descriptive statistics of ratios *r*_1_ (*SCW*/*SCL*), *r*_2_ (*BD*/*SCL*), and *r*_3_ (*BD*/*SCW*) for posthatchlings, and *r*_1_ for adults were similar between different regions for all ratios (Table 2). The null hypothesis that ratios come from the same distribution was not rejected for two cases: posthatchling *r*_3_ and adult *r*_1_ (Mann-Whitney U test, *p* > 0.05). The differences between medians of the ratios posthatchling *r*_1_ and posthatchling *r*_2_ were statistically significant (Mann-Whitney U test, *p* < 0.05), but they were small (1.7% and 1.4% for *r*_1_ and *r*_2_, respectively). Similar descriptive statistics values for ratios of size measurements (Table 2) suggest that the individuals encountered in these two geographic subsets have similar morphology (i.e., shape).

**Table 2:** Descriptive statistics. number of data points (*N*), median, interquartile range (IQR), minimum, and maximum of ratios, for posthatchlings and adults of regional subsets ‘north’ and ‘south’.

posthatchlings	*SCW/SCL*	**N**	**median**	**IQR**	**min**	**max**
	‘south’	1300	0.8308	0.0345	0.5162	0.9784
	‘north’	1267	0.8141	0.0356	0.6345	1.0199
	*BD/SCL*	**N**	**median**	**IQR.**	**min**	**max**
	‘south’	1300	0.4457	0.0274	0.3398	0.5819
	‘north’	1267	0.4378	0.0280	0.3071	0.5727
	*BD/SCW*	**N**	**median**	**IQR**	**min**	**max**
	‘south’	1300	0.5374	0.0419	0.4406	0.8834
	‘north’	1267	0.5395	0.0429	0.3766	0.6450
adults	*SCW/SCL*	**N**	**median**	**IQR**	**min**	**max**
	‘south’	105	0.7638	0.0378	0.6823	0.9268
	‘north’	112	0.7577	0.0414	0.6805	0.9618

Fitting linear scaling models (Eqs 1–3) to the datasets ‘north’, ‘south’, and ‘both’ produced three predictive regression equations (henceforth referred to as ‘*m*_*north*_’, ‘*m*_*south*_’, and ‘*m*_*both*_’) for each model, differing only in parameter values. *R* ^2^ values of all regression equations within the *posthatchling group* were high (0.97 for *SCW*vs*SCL*, and 0.94 for the other two relations, Table 3). Predictive regression equations for one posthatchling dataset showed a small difference in goodness of fit (△*R* ^2^ ⩽ 0.01) when used to describe the other posthatchling dataset (e.g. ‘*m*_*north*_’ models used for the ‘south’ dataset). Likewise, when the general ‘*m*_*both*_’ regression equation was used for describing regional posthatchling data sets (‘south’ or ‘north’), goodness of fit was similar to that of the regionally specific regression equation (△*R* ^2^ ⩽ 0.01). Although slopes (parameter *b*) of some regression equations were statistically different (ANCOVA, *p* < 0.05, Table 3), the width of 95% prediction confidence intervals of ‘*m*_*both*_’ overlaps with that of the subset-specific regression equations (Fig 1 for log(*SCW*) to log(*SCL*) relationship, other relationships not shown but having similar trends). Results within the *adult group* for the log(*SCW*) to log(*SCL*) relationship corroborated the similarity between scaling relationships of different regions. There were again only small differences (△*R* ^2^ ⩽ 0.01) when predictive regression equations for one adult dataset were used for describing the other adult dataset, and there was practically no difference between the *R* ^2^ values of the subset-specific (‘*m*_*north*_’, ‘*m*_*south*_’) and general (‘*m*_*both*_’) regression equations. No two regression slopes within the adult group were significantly different (ANCOVA, *p* < 0.05, Table 3). Considering that we did not find sufficient evidence to support the hypothesis that differences in scaling between ‘north’ and ‘south’ regional subsets are large, we suggest that any analysis can be simplified by grouping the regional subsets.

**Table 3:** Analysis of linear scaling models for regional subsets ‘north’ and ‘south’. For each dataset (listed under ‘datasets’) we analyzed the performance of three predictive regression equations, differing only in the values of model parameters. Parameter values are given under the name of the dataset used for regression. *R* ^2^ value describes the goodness of fit of the regression equation listed in the column to the dataset listed in the row. ‘Slope diff’ indicates whether or not the slopes of two regression equations are significantly different (Tukey-Kramer test, *p* < 0.05), where one regression equation is specific for the dataset listed in the row, and the other for the dataset listed in the column. All regression equations are in the form of *y* = *a* + *b* ⋅ *x* (Eqs 1–3 in ‘Methods’). We analyzed separately data from posthatchlings and adults, see subsection Data for details.

POSTHATCHLINGS					
Scaling	Analysis				
*SCW vs SCL*	**dataset used for regression →**		**‘south’**	**‘north’**	**‘both’**
	**datasets ↓**		*a = -0.3623*	*a = -0.3090*	*a = -0.3303*
			*b = 1.0899*	*b = 1.0523*	*b = 1.0683*
	**‘south’**	R^2^	0.9677	0.9607	0.9659
		Slope diff.	-	**Yes**	**Yes**
	**‘north’**	R^2^	0.9699	0.9769	0.9753
		Slope diff.	**Yes**	-	**Yes**
	**‘both’**	R^2^	0.9689	0.9691	0.9707
		Slope diff.	**Yes**	**Yes**	-
*BD vs SCL*	**dataset used for regression →**		**‘south’**	**‘north’**	**‘both’**
	**datasets ↓**		*a = -0.8124*	*a = -0.7764*	*a = -0.7898*
			*b = 1.0028*	*b = 0.9746*	*b = 0.9863*
	**‘south’**	R^2^	0.9420	0.9349	0.9401
		Slope diff.	-	**Yes**	No
	**‘north’**	R^2^	0.9413	0.9481	0.9464
		Slope diff.	**Yes**	-	No
	**‘both’**	R^2^	0.9416	0.9417	0.9434
		Slope diff.	No	No	-
*BD vs SCW*	**dataset used for regression →**		**‘south’**	**‘north’**	**‘both’**
	**datasets ↓**		*a = -0.4509*	*a = -0.4616*	*a = -0.4564*
			*b = 0.9034*	*b = 0.9092*	*b = 0.9064*
	**‘south’**	R^2^	0.9384	0.9383	0.9384
		Slope diff.	-	No	No
	**‘north’**	R^2^	0.9353	0.9354	0.9353
		Slope diff.	No	-	No
	**‘both’**	R^2^	0.9368	0.9368	0.9368
		Slope diff.	No	No	-
ADULTS					
Scaling	Analysis				
*SCW vs SCL*	**dataset used for regression →**		**‘south’**	**‘north’**	**‘both’**
	**datasets ↓**		*a = 0.7305*	*a = 0.7810*	*a = 0.7193*
			*b = 0.7785*	*b = 0.7681*	*b = 0.7813*
	**‘south’**	R^2^	0.5382	0.5343	0.5374
		Slope diff.	-	No	No
	**‘north’**	R^2^	0.4500	0.4531	0.4523
		Slope diff.	No	-	No
	**‘both’**	R^2^	0.5143	0.5141	0.5151
		Slope diff.	No	No	-

### Analysis of life stage subsets ‘I’, ‘II’, and ‘III’

The standard descriptive statistics of ratios *r*_1_ (*SCW*/*SCL*), *r*_2_ (*BD*/*SCL*), and *r*_3_(*BD*/*SCW*) suggest that morphology of loggerhead turtles differs between life stages. Interquartile range (IQR) of the ratios related to subset ‘I’ was smaller than IQR of the ratios related to subsets ‘II’ and ‘III’ (Table 4), indicating that variability of data in subsets ‘II’ and ‘III’ is larger, even though there are fewer data points than in subset ‘I’. The null-hypothesis that all samples of the same ratio (e.g. $r_{1}^{I}$, $r_{1}^{II}$, and $r_{1}^{III}$) come from the same distribution was rejected (Kruskall Wallis test, *p* < 0.05), however pairwise ratio analysis couldn’t reject the hypothesis that ratios related to subset ‘II’ come from the same distribution as those related to subset ‘III’(e.g. $r_{1}^{II}$, and $r_{1}^{III}$) (Mann-Whitney U test, *p* > 0.05). This was the case for all three ratios, suggesting that subsets ‘II’ and ‘III’ could be merged into a single dataset when applying morphometric scaling, but subset ‘I’ should be considered separately.

**Table 4:** Descriptive statistics. Number of data points (*N*), median, interquartile range (IQR), minimum, and maximum of ratios, for life stage subsets ‘I’, ‘II’, and ‘III’.

*SCW/SCL*	**N**	**median**	**IQR**	**min**	**max**
‘I’	1113	0.823	0.0385	0.510	1.020
‘II’	71	0.819	0.0537	0.741	0.914
‘III’	252	0.761	0.0438	0.680	0.980
*BD/SCL*	**N**	**median**	**IQR**	**min**	**max**
‘I’	1120	0.442	0.0279	0.288	0.521
‘II’	71	0.407	0.0407	0.262	0.485
‘III’	154	0.364	0.0390	0.301	0.549
*BD/SCW*	**N**	**median**	**IQR**	**min**	**max**
‘I’	1112	0.537	0.0409	0.377	0.695
‘II’	59	0.492	0.0583	0.313	0.586
‘III’	147	0.477	0.0531	0.330	0.770

Fitting linear scaling models (Eqs 1–3) to the datasets ‘I’, ‘II’, and ‘III’ produced three predictive regression equations (henceforth referred to as ‘*m*_*I*_’, ‘*m*_*II*_’, and ‘*m*_*III*_’) for each model, differing only in parameter values. For models describing the scaling relationships of carapace width to carapace length (Eq 1), and body depth to carapace length (Eq 2), the slopes (parameter *b*) of ‘*m*_*I*_’, ‘*m*_*II*_’, and ‘*m*_*III*_’ were not significantly different when datasets describing sequential life stages were used for model fitting (Tukey-Kramer test, *p* > 0.05, Table 5 and Fig 2). The relationship of body depth to carapace width (Eq 3) showed a different trend, with the slopes of ‘*m*_*III*_’ significantly different from other slopes (Tukey-Kramer test, *p* < 0.05, Table 5 and Fig 2). In general, ‘*m*_*III*_’ regression equations had the lowest *R* ^2^ values (Table 5), and the widest 95% confidence intervals of parameters and predictions (Fig 2).

**Table 5:** Analysis of linear scaling models for life stage datasets. For each dataset (listed under ‘datasets’) we analyzed the performance of six predictive regression equations, differing only in the values of model parameters. Parameter values are given under the name of the dataset used for regression. *R* ^2^ value describes the goodness of fit of the regression equation listed in the column to the dataset listed in the row. We marked for readability *R* ^2^ values when the regression equation was used for the dataset it was fitted on. ‘Slope diff’ indicates whether or not the slopes of two regression equations are significantly different (Tukey-Kramer test, *p* < 0.05), where one regression equation is specific for the dataset listed in the row, and the other for the dataset listed in the column. All regression equations are in the form of *y* = *a* + *b* ⋅ *x* (Eqs 1–3 in Models and statistical analysis). See subsection Data for definitions.

*SCW vs SCL*	**dataset used for regression →**		**‘I’**	**II’**	**‘III’**	**‘I + II’**	**‘II + III’**	**‘I + II + III’**
	**datasets ↓**		*a = -0.2456*	*a = 0.1072*	*a = 0.5041*	*a = -0.2193*	*a = 0.4014*	*a = -0.1658*
			*b = 1.0233*	*b = 0.9253*	*b = 0.8293*	*b = 1.0094*	*b = 0.8521*	*b = 0.9816*
	**‘I’**	*R* ^2^	0.9902	0.8148	0.0760	0.990	0.3377	0.9886
		Slope diff.	-	No	**Yes**	No	**Yes**	**Yes**
	**‘II’**	*R* ^2^	0.7815	0.8931	0.8805	0.8750	0.8872	0.7885
		Slope diff.	No	-	No	No	No	No
	**‘III’**	*R* ^2^	N/A^†^	0.2053	0.5059	N/A^†^	0.5054	0.4046
		Slope diff.	**Yes**	No	-	**Yes**	No	**Yes**
	**‘I + II’**	*R* ^2^	0.9957	0.9313	0.6587	0.9960	0.7553	0.9952
		Slope diff.	No	No	**Yes**	-	**Yes**	**Yes**
	**‘II + III’**	*R* ^2^	0.5550	0.9021	0.9328	0.7435	0.9335	0.9110
		Slope diff.	**Yes**	No	No	**Yes**	-	**Yes**
	**‘I + II + III’**	*R* ^2^	0.9958	0.9798	0.9023	0.9971	0.9299	0.9982
		Slope diff.	**Yes**	No	**Yes**	**Yes**	**Yes**	-
*BD vs SCL*	**dataset used for regression →**		**‘I’**	**II’**	**‘III’**	**‘I + II’**	**‘II + III’**	**‘I + II + III’**
	**datasets ↓**		*a = -0.7415*	*a = -0.3128*	*a = -0.2475*	*a = -0.7365*	*a = -0.2660*	*a = -0.7075*
			*b = 0.9599*	*b = 0.8525*	*b = 0.8367*	*b = 0.9572*	*b = 0.8408*	*b = 0.9422*
	**‘I’**	*R* ^2^	0.9822	0.6550	0.5443	0.9822	0.5792	0.9819
		Slope diff.	-	No	No	No	**Yes**	No
	**‘II’**	*R* ^2^	0.6860	0.6992	0.6989	0.6885	0.6990	0.6576
		Slope diff.	No	-	No	No	No	No
	**‘III’**	*R* ^2^	N/A^†^	0.1910	0.1942	N/A^†^	0.1942	0.1646
		Slope diff.	No	No	-	No	No	No
	**‘I + II’**	*R* ^2^	0.9918	0.8662	0.8237	0.9918	0.8371	0.9915
		Slope diff.	No	No	No	-	**Yes**	No
	**‘II + III’**	R^2^	0.7512	0.8088	0.8093	0.7626	0.8093	0.7974
		Slope diff.	**Yes**	No	No	**Yes**	-	**Yes**
	**‘I + II + III’**	*R* ^2^	0.9949	0.9476	0.9315	0.9950	0.9366	0.9953
		Slope diff.	No	No	No	No	**Yes**	-
*BD vs SCW*	**dataset used for regression →**		**‘I’**	**II’**	**‘III’**	**‘I + II’**	**‘II + III’**	**‘I + II + III’**
	**datasets ↓**		*a = -0.5072*	*a = 0.1245*	*a = 1.9431*	*a = -0.5234*	*a = -0.3780*	*a = -0.5397*
			*b = 0.9356*	*b = 0.7809*	*b = 0.3772*	*b = 0.9453*	*b = 0.9177*	*b = 0.9549*
	**‘I’**	*R* ^2^	0.9776	0.0068	N/A^†^	0.9775	0.9076	0.9771
		Slope diff.	-	No	**Yes**	No	No	No
	**‘II’**	*R* ^2^	0.4931	0.5755	N/A^†^	0.5397	0.5310	0.5455
		Slope diff.	No	-	**Yes**	No	No	**Yes**
	**‘III’**	*R* ^2^	N/A^†^	N/A^†^	0.0596	N/A^†^	N/A^†^	N/A^†^
		Slope diff.	**Yes**	**Yes**	-	**Yes**	**Yes**	**Yes**
	**‘I + II’**	*R* ^2^	0.9892	0.5963	N/A^†^	0.9893	0.9610	0.9892
		Slope diff.	No	No	**Yes**	-	No	No
	**‘II + III’**	*R* ^2^	0.6931	0.6521	0.3818	0.7322	0.7512	0.7489
		Slope diff.	No	No	**Yes**	No	-	No
	**‘I + II + III’**	*R* ^2^	0.9938	0.8562	N/A^†^	0.9941	0.9845	0.9943
		Slope diff.	No	**Yes**	**Yes**	No	No	-

^†^ The linear model *y = a + bx* underperforms relative to the null-model with *b = 0*.

**Fig 3:**
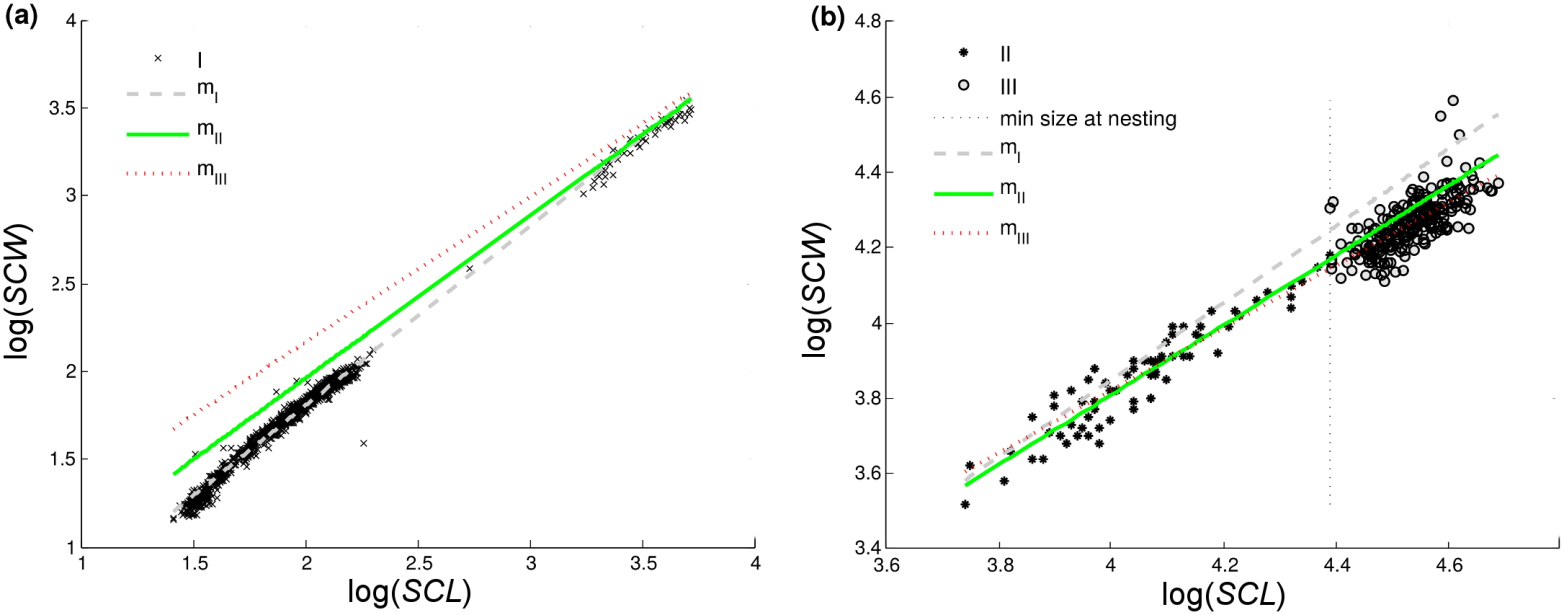
Predictions of log(*SCW*) by regression equations ‘*m*_*I*_’, ‘*m*_*II*_’, and ‘*m*_*III*_’. Regression equations are specific for life stage subsets ‘I’, ‘II’, and ‘III’ (respectively). Panel (a): subset ‘I’, panel (b): subsets ‘II’ and ‘III’. Parameters for the equations are provided in Table 5.

We then used predictive regression equations specific to one subset, to predict values for the other two subsets. Results are given in Table 5 for all three scaling relationships, and in Fig 3 for the relationship of carapace width to carapace length (Eq 1). Predictions for the relationship of body depth to carapace length (Eq 2) were similar to the ones shown in the figure: only ‘*m*_*I*_’ was suitable for subset ‘I’, and only ‘*m*_*II*_’ and ‘*m*_*III*_’ described subsets ‘II’ and ‘III’ with satisfactory accuracy. For the relationship of body depth to carapace width (Eq 3), again only ‘*m*_*I*_’ was suitable for subset ‘I’, however the slopes (Tukey-Kramer test, *p* < 0.05) and consequently predictions of ‘*m*_*II*_’ and ‘*m*_*III*_’ were significantly different. This might have been a consequence of data scatter in subset ‘III’, and relatively low correlation of log *BD* to log *SCW* (*R* ^2^ = 0.06) (Table 5).

**Fig 4:**
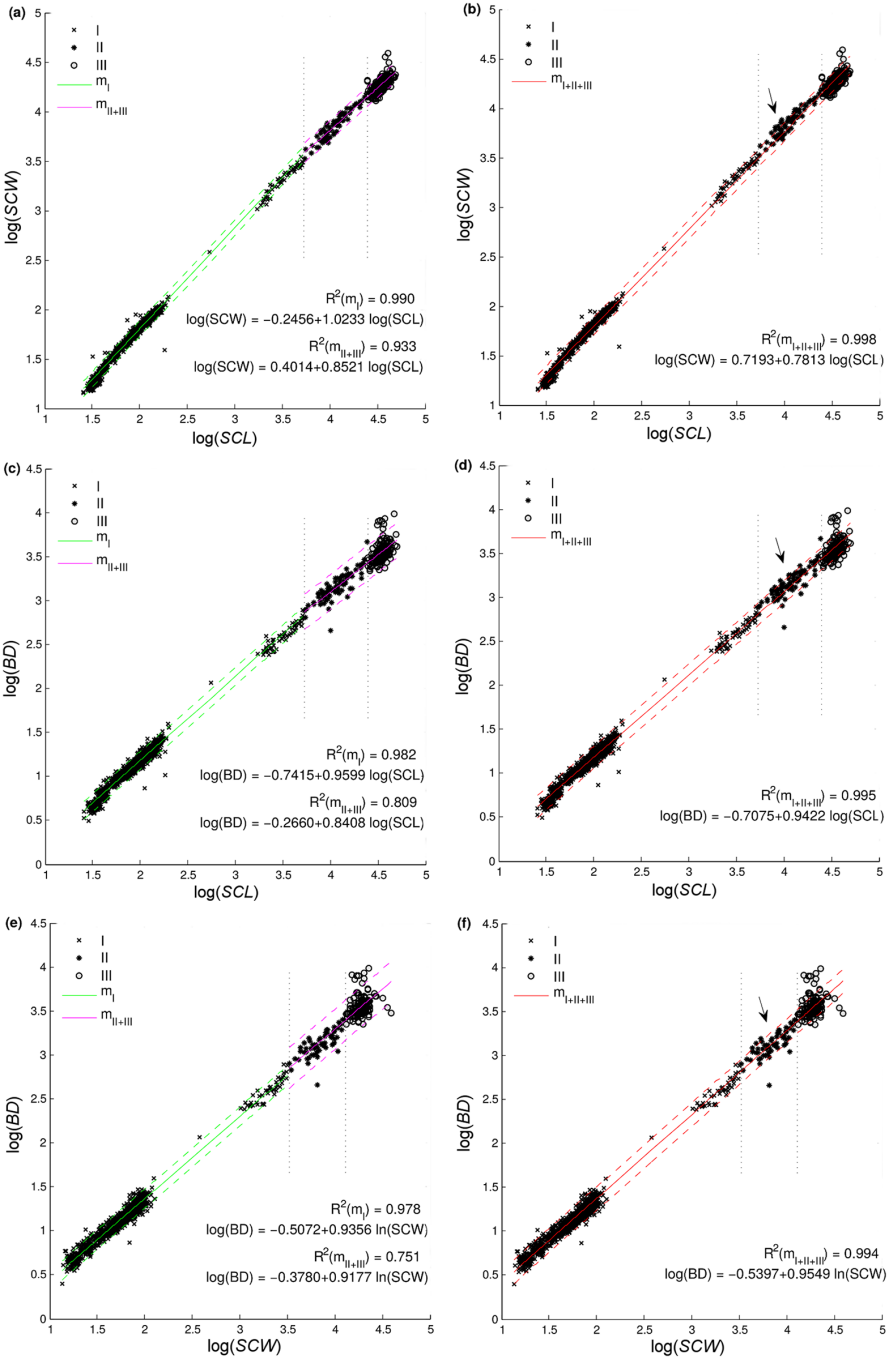
Fit of suggested subset-specific (‘*m*_*I*_’, ‘*m*_*II* + *III*_’, panels (a), (c), (e)), and non-specific (‘*m*_*I* + *II* + *III*_’, panels (b), (d), (f)) linear scaling models to data. The relationship of log(*SCW*) to log(*SCL*) is shown in panels (a) and (b), the relationship of log(*BD*) to log(*SCL*) in panels (c) and (d), and the relationship of log(*BD*) to log(SCW) in panels (e) and (f). The recommended regression equations are displayed in the plot, while parameters for remaining equations are provided in Table 5. Dashed lines mark the 95% confidence intervals of the predictions. Black arrows in panels (b), (d), and (f) point to the size range in which predictions are underestimated.

When we analyzed the performance of models fitted on combined datasets (‘I + II’, and ‘II + III’), the slopes of predictive regression equations ‘*m*_*I* + *II*_’ and ‘*m*_*II* + *III*_’ were not significantly different from the slopes of predictive equations for the subsets merged into the combined datasets (Tukey-Kramer test, *p* > 0.05, Table 5). However, ‘*m*_*I* + *II*_’ slightly overestimated log(*SCW*) and log(*BD*) for larger individuals in subset ‘II’, and the relatively narrow 95% prediction confidence interval of ‘*m*_*I* + *II*_’ could not account for the increase of data scatter in subset ‘II’. This suggests that ‘*m*_*I* + *II*_’ is not suitable for modeling large neritic juveniles. By contrast, 95% prediction confidence intervals of ‘*m*_*II* + *III*_’ are very similar to those of ‘*m*_*II*_’ and ‘*m*_*III*_’, giving a wide enough range for predictions, and *R* ^2^ value of ‘*m*_*II* + *III*_’ for subsets ‘II’ and ‘III’ was practically the same as *R* ^2^ values of ‘*m*_*II*_’ and ‘*m*_*III*_’ for those subsets (Table 5). Model analysis, therefore, supports the idea that life stage subsets ‘II’ and ‘III’ (representing neritic juveniles and adults) can be merged. Description and predictions of models that we suggest should be used for loggerhead turtles are given in Fig 4, panels (a), (c), and (e).

Next, we fitted models (1–3) to the dataset combining all three subsets (‘I + II + III’), and obtained predictive regression equations ‘*m*_*I* + *II* + *III*_’. Description and predictions of ‘*m*_*I* + *II* + *III*_’ are given in Fig 4, panels (b), (d), and (f). Although predictions were satisfactory over the whole size span, some values for juveniles in the subset ‘II’ were underestimated, which is consistent with the gradual change (decrease) in model slope in later life stages. Generally however, *R* ^2^ values of ‘*m*_*I* + *II* + *III*_’ for subsets ‘I’, ‘II’, and ‘III’ were as high as those of ‘*m*_*I*_’, ‘*m*_*II*_’, and ‘*m*_*III*_’ (Table 5), suggesting that this model can be applied for describing the whole size span of loggerhead turtles.

Finally, we analyzed whether the growth of loggerhead turtles can be considered isometric. The slope coefficients of ‘*m*_*I* + *II* + *III*_’ were significantly different from 1 (Tukey-Kramer test, *p* < 0.05) for all studied relationships (Fig 2), suggesting allometric growth. However, the differences between values calculated by the allometric linear model (regressed value of *b*) and the isometric linear model (*b* = 1) for the same relationship were less than 5%. Furthermore, the predictions of the isometric model were within the range of predictions of the allometric model for the same relationship (Table 6).

**Table 6:** Comparison of the allometric model (value of *b* regressed by model fitting) to the isometric model (*b* = 1). As the allometric model we used the predictive regression equation ‘*m*_*I* + *II* + *III*_’. As size at the event of interest, we used average values at hatching SCL = 4.5 cm [33], recruitment SCL = 48 cm [9], and nesting SCL = 93 cm [28, 31] for the relationships of carapace width and body depth to carapace length. For the relationship of body depth to carapace width we calculated SCW values that would correspond to average carapace lengths at hatching, recruitment, and nesting, using ‘*m*_*I* + *II* + *III*_’. Error was calculated for log_*e*_ transformed data as [100(value predicted by isometric model—value predicted by allometric model)/ value predicted by allometric model].

			predictions range (cm)	
event of interest	relationship	error (%)	*allometric model*	*isometric model*
	*SCW vs SCL*	-1.41	3.39–4.06	3.63–3.65
hatching	*BD vs SCL*	-7.05	1.79–2.30	1.92–1.94
	*BD vs SCW*	-5.17	1.78–2.33	1.95–1.97
	*SCW vs SCL*	0.69	34.60–41.45	38.73–38.93
recruitment	*BD vs SCL*	2.95	16.69–21.43	20.53–20.72
	*BD vs SCW*	2.32	16.37–21.45	19.97–20.14
	*SCW vs SCL*	0.87	66.23–79.33	75.04–75.43
nesting	*BD vs SCL*	3.51	31.12–39.98	39.78–40.15
	*BD vs SCW*	2.74	30.42–39.87	38.22–38.55

### Performance of saturating models ‘M2’

**Fig 5:**
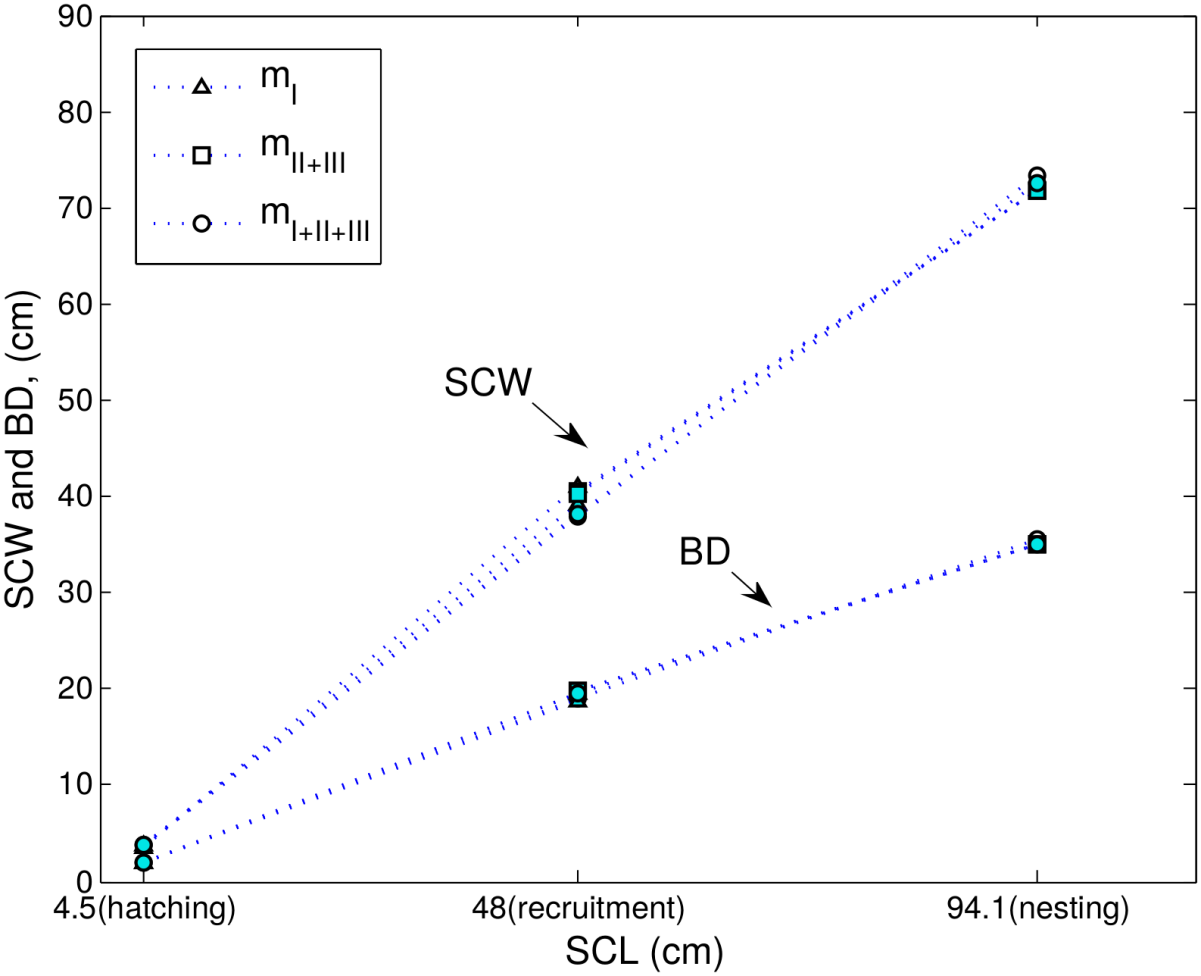
Predictions of SCW and BD by two types (M1—linear, and M2—saturating) of models ‘*m*_*I*_’, ‘*m*_*II* + *III*_’, and ‘*m*_*I* + *II* + *III*_’. Predictions are given for average sizes at specific events (hatching, recruitment, nesting). Symbols are coded based on the model (each symbol corresponds to one model), and type (full or empty symbol).

Saturating (curvilinear) models,‘M2’, (Eqs 4–6) did not perform markedly better than the commonly used linear models, ‘M1’, (Eqs 1–3). We tested all models on data sets ‘I’, ‘II + III’, and ‘I + II + III’ that, based on the previous analysis, need to be taken into the account for morphometric scaling. Models ‘M1’ and ‘M2’ have satisfactory and almost identical goodness of fit (*R* ^2^ values, RMSE) for all relationships of all data sets taken into account (Table 7, Fig 5). Even though Akaike weights in some cases point with a 100% certainty to a certain model, predictions between the linear and nonlinear class of the same model differ 0.0153—4.5093% for SCW and 0.0029—4.6456% for BD.

**Table 7:** Comparison of the linear (lin.) and saturating (sat.) type of models ‘*m*_*I*_’, ‘*m*_*II* + *III*_’, and ‘*m*_*I* + *II* + *III*_’ for the three studied relationships. Performance of models was tested on datasets ‘I’, ‘II + III’, and ‘I + II + III’, and evaluated by goodness of fit statistics (*R^2^*, Root Mean Square Error (RMSE)), and Akaike weights).

dataset/*model*	type of model	log *(SCW)* vs log *(SCL)*			log *(BD)* vs log *(SCL)*			log *(BD)* vs log *(SCW)*		
		*R^2^*	*RMSE*	*Akaike weight*	*R^2^*	*RMSE*	*Akaike weight*	*R^2^*	*RMSE*	*Akaike weight*
**‘I’/**‘*m*_*I*_’	lin.	0.9902	0.0391	1.0000	0.9822	0.0519	0.0000	0.9776	0.0557	1.0000
	sat.	0.9897	0.0401	0.0000	0.9828	0.0510	1.0000	0.9763	0.0572	0.0000
**‘II + III’/**‘*m*_*II* + *III*_’	lin.	0.9335	0.0492	0.2856	0.8093	0.1003	0.5525	0.7512	0.1149	0.3647
	sat.	0.9339	0.0491	0.7144	0.8090	0.1004	0.4475	0.7525	0.1146	0.6353
**‘I + II + III’/**‘*m*_*I* + *II* + *III*_’	lin.	0.9982	0.0460	0.0000	0.9953	0.0637	0.0000	0.9943	0.0688	1.0000
	sat.	0.9983	0.0439	1.0000	0.9955	0.0623	1.0000	0.9940	0.0704	0.0000

## Discussion

We analyzed morphometric scaling relationships for straight carapace width (SCW), straight carapace length (SCL), and body depth (BD) of loggerhead turtles using all available data for the western North Atlantic population. The analysis included the scaling relationships of two regional (‘north’, ‘south’), and three life stage (‘I’, ‘II’, ‘III’) subsets, as well as two types of the scaling models: linear and saturating.

Our results suggest that the following models can be used to reasonably well describe scaling relationships of all western North Atlantic loggerhead turtles (all *R* ^2^ > 0.99), natural logarithm was used:

log(SCW)=-0.1658+0.9816·log(SCL),

log(BD)=-0.7075+0.9422·log(SCL),and

log(BD)=-0.5397+0.9549·log(SCW).

Earlier analysis [14] gave similar relationships, especially for the relationship of carapace width and carapace length (log *SCW* = −0.0225 + 0.9507 ⋅ log *SCL*, and log *BD* = −0.5682 + 0.9100 ⋅ log *SCL*). Our analysis is (i) based on considerably more (>6x) data, and (ii) represents a broader size range (>120x more data for turtles with SCL<20cm and SCL>100cm). Data for sea turtles at the lower end of the size range are mostly obtained from captive reared turtles (here, and in [14]). The scaling relationships of width to length are presumed not to be affected by rearing conditions, but this does present a potential source of error in our data. Pooling captive-reared and wild data, as well as individual variability, may increase data scatter and model uncertainty. Having data from various sources, with potential differences in measurement accuracy and precision add additional sources of scatter. Individual variation has been reported for body depth [33, 36], and dependence of BD measurements on sea turtle’s inhalation/exhalation adds accuracy and precision error. In most cases captive reared turtles are fed *ad libitum* to maximize growth, and BD will be greater in these captive data in relation to their wild counterparts. Consequently, the largest scatter was found in the BD data.

Our results suggest that analyzing different life stages of loggerhead turtles separately is appropriate when accuracy is important. For young loggerhead turtles that have not recruited to neritic habitats (posthatchlings and oceanic juveniles smaller than 41.5 cm SCL [9]), predictive regression equations are:

$$\log\left(SCW\right)=-0.2456+1.0233\cdot \log\left(SCL\right),R^{2}=0.9902,$$

$$\log\left(BD\right)=-0.7415+0.9599\cdot \log\left(SCL\right),R^{2}=0.9822,and$$

$$\log\left(BD\right)=-0.5072+0.9356\cdot \log\left(SCW\right),R^{2}=0.9776.$$

For neritic juveniles and nesting adults (loggerhead turtles larger than 41.5 cm SCL), we recommend:

$$\log\left(SCW\right)=0.4014+0.8521\cdot \log\left(SCL\right),R^{2}=0.9335,$$

$$\log\left(BD\right)=-0.2660+0.8408\cdot \log\left(SCL\right),R^{2}=0.8093,and$$

$$\log\left(BD\right)=-0.3780+0.9177\cdot \log\left(SCW\right),R^{2}=0.7512.$$

Special attention should be given when using relationships to estimate BD for adults: correlation of BD to SCW and SCL for adults is extremely weak (*R* ^2^ = 0.06, and 0.19 respectively). This is presumably a consequence of large scatter in BD data, more pronounced for large individuals.

### Regional subsets

When studying the scaling relationships of SCW and BD to SCL, our results suggest that regional models (‘*m*_*north*_’ and ‘*m*_*south*_’) fitted to one regional subset can be used to describe the other, and the models fitted on both subsets (‘*m*_*both*_’) describe each of the regional subsets with satisfactory accuracy (negligible differences in *R* ^2^ value, overlapping 95% prediction confidence intervals). Statistical differences between the model slope of the ‘*m*_*both*_’, and slopes of the regional models were found only for the log(*SCW*) to log(*SCL*) relationship within the posthatchling group. This might be a consequence of regionally-specific growth rates in the first couple of weeks, or different rearing conditions: water temperature and food availability [33]. Because of the large sample size (*N* > 1500, this might be the case when “highly significant statistics do not signify equally high biological significance” [12].

Although previous research suggested that regional differences in scaling could be important between the same regions as analyzed here [28, 36], we claim that the differences are negligible. The results of our analysis were not considerably different regardless of whether the subsets were analyzed together or separately, and we suggest that any analysis can be simplified by grouping regional subsets (sea turtles belonging to the same life stage encountered in the specific geographic area) of the western North Atlantic. [28] computed scaling relationships of carapace width and body depth to carapace length for 130 loggerhead turtles nesting in South Carolina, and found significant differences between the scaling relationship of SCW to SCL and the same relationship published for Florida turtles in [32]. Ref. [28] suggested that the differences in scaling relationships could be caused by regional conformational differences, but noted that other causes (e.g. sampling differences and/or allometric growth) could be responsible. Our analysis, comparing individuals of similar sizes and life stages, did not detect considerable differences between regions. We therefore suggest that the differences observed by [28] are not regional, but instead should be attributed to other causes. This is supported by the findings that SCL and SCW are uniform across the western North Atlantic for nesting females [36]. The latter analysis, however, found a decrease in BD along the north-to-south gradient, not mentioned in [28]. Taking into account the uniform SCL and SCW reported in the same publication [36], the gradual change in BD should be reflected in regionally-specific scaling relationships that include BD (relationships of body depth to carapace length and to carapace width), with a steeper slope of the model fitted to the northern subset. Due to lack of data, we could not test for differences of scaling relationships that include BD within the adult group, but we could analyze the relationships within the posthatchling group. Surprisingly, the analysis suggested that, for the relationship of body depth to carapace length, it is more likely that the slope of the model specific for the **south** subset will be steeper, suggesting an increase of body depth along the north-to-south gradient. However, as mentioned earlier, scatter in BD data is high. Different measurement techniques and precision, as well as different conditions sea turtles were exposed to prior to the measurements, certainly introduced a substantial error and uncertainty. Body depth may vary by season, as water temperatures and prey quality and availability fluctuate. Additionally, BD may fluctuate from the beginning to the end of the nesting season, as females often don’t feed during the nesting period [40]. So, comparing a BD measurement taken at the beginning of the season in one region, to one taken at the end of the season in another region might produce a misleading result. More standardized measurements should be obtained for any relevant conclusions.

### Life stage subsets

The analysis among the three life stages revealed significant differences in growth of width and depth relative to body length. Differences between *sequential* life stages were not significant for any of the studied relationships, suggesting a gradual, cumulative life-long allometric growth. Differences between subsets ‘I’ (posthatchlings and oceanic juveniles) and ‘III’ (nesting adults) were significant for scaling of SCW and BD to SCL; the analysis of the differences in BD relative to SCW were inconclusive because of a large scatter in BD measurements for adults. The decrease in slope of models (Eqs 1 and 2) for SCW and BD as a function of increasing SCL (Fig 2) is consistent with such a gradual process. Allometric growth changes throughout the life: initially, SCW and BD out-grow SCL; later in life, SCL grows faster than either SCW or BD. Therefore, extrapolating scaling from one life stage onto another should be avoided. Caution with such extrapolations was suggested before [29], but was not studied in more detail. To study the change in allometric growth, more data on loggerhead turtles in the critical range (between 10 and 25 cm SCL) is necessary.

When extrapolated, predictive regression equations for subset ‘I’ overestimate carapace widths and body depths for subsets ‘II’ (neritic juveniles) and ‘III’ (Fig 3). When describing scaling in turtles, we recommend using one set of regression equations (models ‘*m*_*I*_’) for posthatchlings and oceanic juveniles (young sea turtles before recruitment, at SCL<41.5cm, [9]), and another set of equations (‘*m*_*II* + *III*_’) for neritic juveniles and adults.

Special attention is needed when predicting values for BD of adults. Correlations of BD to SCW and SCL are weak (*R* ^2^ of 0.06 and 0.19, respectively). The weak correlation is in large part because of high scatter of available BD data, possibly due to regional gradients in BD observed previously [36]. Additional sources of scatter of BD measurements could be individual variability, different environmental conditions that influence growth, such as temperature and food availability [41], and lack of standardized measurement techniques (e.g. unambiguous reference points on the turtle carapace) that could be applied when measuring body depth [30, 41]. Consequently, certainty of predictive regression equations is low, and using scaling relationships to predict BD in adults should be avoided whenever possible. Grouping neritic juveniles and adults reduces variability and increases confidence in predictions; the grouped model describes SCW well (*R* ^2^ > 0.93), and yields acceptable predictions for BD (*R* ^2^ > 0.8). Predictions of the allometric and isometric models were not considerably different, suggesting that the growth of sea turtles is close to being isometric.

### Saturating models

Based on the results of data exploration, we tested the performance of saturating scaling models. The tested models did not perform considerably better than linear, and predictions of the linear and saturating model for the same scaling relationship differed less than 4.7%. Surprisingly, for some data that showed the most pronounced curvilinear trend when plotted on log-log axes, the linear model was statistically preferred: e.g. the SCW to SCL relationship for posthatchlings in subset ‘I’ (see Fig 1, panels (a,b) and Table 7). In general, the results suggest that it is much more important to use the appropriate model (e.g. ‘*m*_*II* + *III*_’ for neritic juveniles and/or adults), than it is to use the statistically preferred class of the model (linear or saturating). The linear models account for >90% data variance (Table 5), so the result is not surprising. The additional complexity of the saturating models is not justified from practical aspect, and their use will probably be limited. However, even though the linear models may be sufficiently accurate for most applications, the significant difference between morphometric scaling of different life stages found here might benefit from further research [13]. Curvilinear models, such as the saturating models tested here or the models suggested by other authors [37, 38], could prove to be more appropriate than the linear model when accuracy is of utmost importance.

### Implications

Scaling relations do not describe the mechanisms or causes of correlation of the observed variables, but they are helpful for discovering patterns, and obtaining predictive regression equations.

Similarity of morphological scaling relationships among different regional subsets of western North Atlantic loggerheads justifies the use of the same equation set for more than one specific subset, or for a whole population. For a theoretical example, if we assume that juveniles and adults in a neritic area are affected by shrimp trawls, we can use the ‘*m*_*II* + *III*_’ model to estimate the lower boundary of the TED opening size. For the sake of illustration, if we assume a uniform size distribution (which we know to be biased because an actual population would generally have a declining density distribution with size), we can look at the 97-percentile of the size range because the targeted 97% TED efficiency in part depends on the size structure of a population in an area. Using *SCL*_*min*_ = 41.5 cm (minimum size at recruitment, [9]), and *SCL*_*max*_ = 130 cm (largest known nesting female, [42]), the 97-percentile of this theoretical size range is 127 cm. This corresponds to a SCW of 93.3 cm, and BD of 45.3 cm (predicted by regression equations ‘*m*_*II* + *III*_’ specific for neritic juveniles and adults). TED opening—from the size considerations alone—should therefore be a minumum of 93.3 x 45.3 cm. However, although such theoretical size estimates are important for evaluating TED opening dimensions, these are based on just one species (loggerhead turtles), and do not take into account the causes of entanglement for reasons not related to size. When evaluating TED design and dimensions, National Marine Fisheries Service (NMFS) targets 97% efficiency in excluding sea turtles during experimental TED testing, accounting for factors such as: angle of installation, debris, fouling, and other issues, and required dimensions account also for the largest possible individuals, including large green and leatherback turtles ([43], Vol 68, No 35). Current regulations of design and dimensions of TED openings in North Atlantic [44] appear conservative enough to exclude loggerhead turtles based on our analysis. Predictive regression equations reported in this study for loggerhead turtles may be informative in future evaluations.

Scaling relations of a single species’ size measurements describe the current shape of the animals, and should not be used for explaining evolutionary or other processes [13]. However, scaling is a result of physical principles and various ecological/biological factors [45], and can be a valid starting point for exploring the underlying processes. For example, difference in scaling relationships between posthatchlings and other (later) life stages could reflect constraints faced by the sea turtles in different life stages. Sea turtles could be initially growing preferentially in width to avoid predators [18], and in length later in life to increase in size and improve hydrodynamics, chances of survival [17, 18], and fecundity [46]. Alternatively, smaller oceanic turtles become large enough to capably exploit neritic habitats, and undergo an ontogenetic shift to coastal waters at a threshold carapace size [5]. However, different regression routines should be applied for further analysis of the (non-significant) decrease of the slope throughout the life cycle, and interspecific morphometric analysis should be conducted in order to make conclusions involving evolutionary preferred solutions (see [13] for overview of analyses in scaling).

We can use the slope of a scaling relationship not only to compare one relationship to another, but also to infer whether or not the organism’s growth is isometric. As mentioned before, the differences between scaling relationships of sequential life stage subsets were not significant, and one set of scaling relationships (marked as ‘*m*_*I* + *II* + *III*_’) described well the whole analyzed size span of loggerhead turtles, for all three analyzed relationships (SCW to SCL, BD to SCL, and BD to SCW). The comparison of predictions by the current (allometric) models to the predictions by the isometric models (with *b = 1*), suggests that the growth of loggerhead turtles can be considered isometric without losing much accuracy. Additionally, the changes in scaling may not affect the growth of total body volume; for example, losses of volume due to slower growth in SCW could be offset by faster growth in SCL. This is supported by the reported scaling of mass to length to the power close to 3 [22] (mass scaling to cubed length would be isometric scaling). In case of isometric growth, i.e., when proportional relationships are preserved while size changes during ontogeny or evolution, a set of implied properties can be applied: all volume-based properties scale proportionally to volume (often expressed as body mass), all surface area-based properties scale with volume to the power 2/3, and all length-based properties scale with volume to the power 1/3 [12, 47]. Further simplification can be made by assuming that the physical length is always the same fraction of the volumetric length (volume to the power 1/3), relating length-based properties directly to physical length. The slight offset from isometric scaling that has been detected needs to be kept in mind, but the (mostly) isometric growth opens the door to simplifications in a variety of modeling applications.
